# Isoproterenol Increases Left Atrial Fibrosis and Susceptibility to Atrial Fibrillation by Inducing Atrial Ischemic Infarction in Rats

**DOI:** 10.3389/fphar.2020.00493

**Published:** 2020-04-15

**Authors:** Shiyu Ma, Jin Ma, Qingqiang Tu, Chaoyang Zheng, Qiuxiong Chen, Weihui Lv

**Affiliations:** ^1^ The Second Affiliated Hospital of Guangzhou University of Chinese Medicine, Guangdong Provincial Hospital of Chinese Medicine, Guangzhou, China; ^2^ Zhongshan School of Medicine, Sun Yat-sen University, Guangzhou, China

**Keywords:** atrial fibrillation, fibrosis, myocardial ischemia, isoproterenol, atrium

## Abstract

Left atrial (LA) fibrosis is a major arrhythmogenic substrate for atrial fibrillation (AF). The purpose of this study was to assess whether isoproterenol (ISO) induces LA fibrosis and increases susceptibility to AF, exploring the underlying mechanisms. Male Sprague-Dawley rats were subcutaneously injected ISO once per day for 2 days. Five weeks after injection, the ISO group had higher susceptibility AF and prolonged AF duration compared with the control group. ISO decreased LA conduction velocity (CV) and increased LA conduction heterogeneity. ISO increased fibrosise areas and the protein levels of collagen types I and III in the left atrium. Antifibrosis drug pirfenidone decreased AF occurrence and reduced LA fibrosis in ISO treated rats. ISO injection induced atrial ischemia infarction by increasing heart rate and decreasing diastolic and systolic blood pressures. These findings demonstrated that ISO increases susceptibility to AF by increasing LA fibrosis and LA conduction abnormalities 5 weeks after injection. ISO injection induces atrial ischemic injury is the main cause of fibrosis. Rats with ISO-induced LA fibrosis may be used in further AF research.

## Introduction

Atrial fibrillation (AF) is the most common tachyarrhythmia; its incidence increases due to widespread population aging. AF is the final common endpoint of atrial remodeling caused by a variety of cardiac diseases and conditions, and promotes important remodeling that contributes to the progressive nature of arrhythmia (Tan and Zimetbaum, 2011). Left atrial (LA) fibrosis is considered the key element of atrial remodeling in patients with structural heart disease and persistent AF (Velagapudi et al., 2013). Experimental studies have provided convincing evidence that fibrotic transformation of the left atrium results in the deterioration of atrial conduction, increasing impulse propagation anisotropy and building boundaries that promote re-entry in the atrial wall, which may be directly relevant for the mechanisms responsible for AF maintenance (Heijman et al., 2014; Krul et al., 2015).

Catecholamines increase the contractile force and beating rate of the heart, resulting in markedly increased cardiac pumping output and oxygen consumption (Nichtova et al., 2012). Excess of catecholamines in circulation is responsible for myocardial tissue damage in clinical conditions such as ischemia, angina, infarction, cardiac arrhythmias and sudden cardiac death. Increased administration of exogenous catecholamines leads to remodeling of myocardium and cardiomyocytes at the subcellular level. Isoproterenol (ISO) is a synthetic catecholamine and nonselective β-adrenoceptor agonist. Single or repeated doses of ISO administered to experimental animals induce fibrosis (Ma et al., 2017), cardiac hypertrophy, and myocardial damage in the left ventricle. ISO models contribute effectively to the understanding of pathologies in signal transduction, energetic, excitability, and contractility, which may contribute concomitantly to cardiac dysfunction and heart failure (Nichtova et al., 2012). The aim of this study was to assess whether ISO could induce LA fibrosis and increase susceptibility to AF and exploring the underlying mechanisms.

## Materials and Methods

### Animals

All animal experiments were performed in accordance with the National Institutes of Health Guidelines for the Care and Use of Laboratory Animals, and the National Standard of the People’s Republic of China for Laboratory animal Guidelines for ethical review of animal welfare. Male Sprague-Dawley rats (9-10 weeks, SPF, Guangdong Medical Experimental Animal Center) were housed at 20 ± 3°C and 55% ± 10% humidity, under a 12-h–12-h light/dark cycle. ISO hydrochloride (Sigma-Aldrich, St. Louis, MO, USA) was dissolved and injected subcutaneously at different doses once daily for two days. Choose the best dose for the animal study. The rats were divided into three groups, including control (CTL), ISO injection (ISO), and ISO injection with pirfenidone (PFD) treatment (ISO+PFD) groups. PFD is a broad-spectrum antifibrotic drug that has shown potential in numbers of animal models of fibrosis and clinical trials (Lopez-de La Mora et al., 2015). One week after injection, PFD (Sigma-Aldrich, St. Louis, MO, USA) was dissolved in water and gavaged for 4 weeks at a dose of 300 mg/kg in the ISO+PFD group. Meanwhile, equal volume of water was gavaged to the control and ISO groups for 4 weeks.

### 2, 3, 5-Triphenylteyltetrazolium Chloride Staining

The heart was removed and cooled in a −20°C freezer. After freezing, heart sections at 2 mm thickness were obtained. Atria were then incubated with 2% 2, 3, 5-triphenylteyltetrazolium chloride (TTC) (Sigma-Aldrich, St. Louis, MO) in a 37°C bath for 20 min to visualize the unstained infarcted region. After TTC staining, viable myocardium stained brick red while the infarct appeared pale white. TTC-stained tissue sections were photographed using a digital scanner.

### Echocardiogram

After the induction of general anesthesia by 2% sevoflurane.The probe (Vevo 2100 system and MS-250 transducer, VisualSonics Inc, Canada) was placed on the chest and collected data along the short and long axes of the heart in all groups. Signals from M-mode echocardiography were recorded. Parameters obtained from the echocardiogram including left ventricular internal dimensions during systole (LVIDs) and diastole (LVIDd), the ejection fraction (EF), and fractional shortening (FS) were measured according to the leading-edge method. Each echocardiographic variable was determined in at least four separate images taken from the same heart.

### Programmed Electrical Stimulation and Induction of AF

AF was defined as irregular, rapid atrial activation with varying electrogram morphology lasting ≥2 s, as we described previously (Ma et al., 2018a; Ma et al., 2018b). Rats were anesthetized with urethane and instrumented with subcutaneous electrodes for ECG recordings (Power Lab 16/35, AD Instruments, Castle Hill, NSW, Australia). The rat was tracheotomized and ventilated (Harvard Apparatus, Holliston Co) with room air supplemented with oxygen at 65 breaths/min. For atrial stimulation, a 4-French quadripolar catheter was advanced through the esophagus and placed at the site with the lowest threshold for atrial capture. Atrial pacing was performed at twice the diastolic threshold using two poles on the pacing catheter. Inducibility of AF was tested by applying 35-s bursts. The burst had a cycle length of 20 ms and pulse width of 5 ms. This series of bursts was repeated once. All rats were allowed 5 min of recovery in the sinus rhythm between stimulations for respiratory and circulatory recovery. If one or more bursts in the two series of bursts evoked an AF episode, AF was inducible in that rat. Otherwise, AF was noninducible. The duration and probability of inducible AF episodes were analyzed. The longest record time was 30 min after the burst pacing.

### Multielectrodes Arrays Measurements

Multielectrode arrays (MEA) measurements were performed, as we described previously (Ma et al., 2018a; Ma et al., 2018b). The heart was removed rapidly, and the left atrium from the isolated heart was dissected and then immersed in Tyrode’s solution. For MEA mapping, the epicardial LA surface rested on the MEA (Multi Channel Systems, Reutlingen, Germany) culture dish containing 120 tipped platinum recording electrodes of diameter 30 μm with an interelectrode spacing of 100 µm, and continuously superfused at a flow rate of 3 ml/min with oxygenated Tyrode’s solution with at 37°C. During recordings, contractility was blocked with 15 mM butadione monoxime (BDM). The electrode arrays were mounted onto a printed circuit board and then fitted into the MEASystem interface. Electrical stimulation (bipolar pulses, 1–7 V, 1,000-µs duration) was applied *via* one of the MEA microelectrodes. Data were sampled at 10 kHz per channel with simultaneous data acquisition using the Cardio 2D software (Multi Channel Systems), and five fields were recorded in each atrium. All the data were analyzed to generate activation maps and measure CV.

### Masson Trichrome Staining

For the quantification of atrial fibrosis, Masson’s trichrome staining of coronalplane slices prepared from paraformaldehyde fixed samples was performed as previously described (Ma et al., 2017). Slices (5 μm) were stained with Masson’s trichrome, and photographed using a digital camera under a BX53 microscope (Olympus, Tokyo, Japan). Images were quantified by the CellSens Dimension 1.16 software. Fibrotic areas were expressed as a percentage of blue-positive stained area to the total tissue area.

### Western Blot

Protein from samples was separated by SDS-PAGE. Separated protein was transferred on a polyvinylidene difluoride membrane that was blocked at room temperature for 1 h in Tris-buffered saline with 0.2% Tween 20 containing 5% skim milk and probed with primary antibodies overnight at 4°C. Protein bands on Western blot were visualized using ECL Plus (Millipore, Billerica, MA, USA). Relative band densities of proteins were normalized against GAPDH.

### Implantation of Telemetry Transmitter

Seven days prior to the test, a telemetry transmitter (Millar Instruments, Houston, TX, USA) was implanted and secured in the abdominal cavity, with the leads tunneled under the skin. The rats were housed in individual cages placed on a receiver that continuously captured signals, independent of animal activity. The signals were recorded with the LabChart 8 software and stored for analysis.

### Cardiac Marker Enzyme Levels in the Serum

Two hours after the second injection of ISO, collected serum samples were assesssed for the cardiac marker enzyme creatinine kinase-MB (CK-MB). Analysis was performed with commercially available standard enzymatic kits.

### Data Analysis and Statistics

Data were expressed as mean ± SD except for AF duration, which was expressed as median and interquartile range (25%–75%). The Fisher exact test was applied to compare AF inducibility. Normally distributed variables were tested using one-way analysis of variance (ANOVA). Differences between nonnormally distributed variables were examined by Mann-Whitney U test. All data analysis was performed using SPSS statistical software (SPSS, IL, USA). Statistical significance was defined as *P* < 0.05.

## Results

### ISO Injection Causes LA Ischemia and Fibrosis

**Figure 1:**
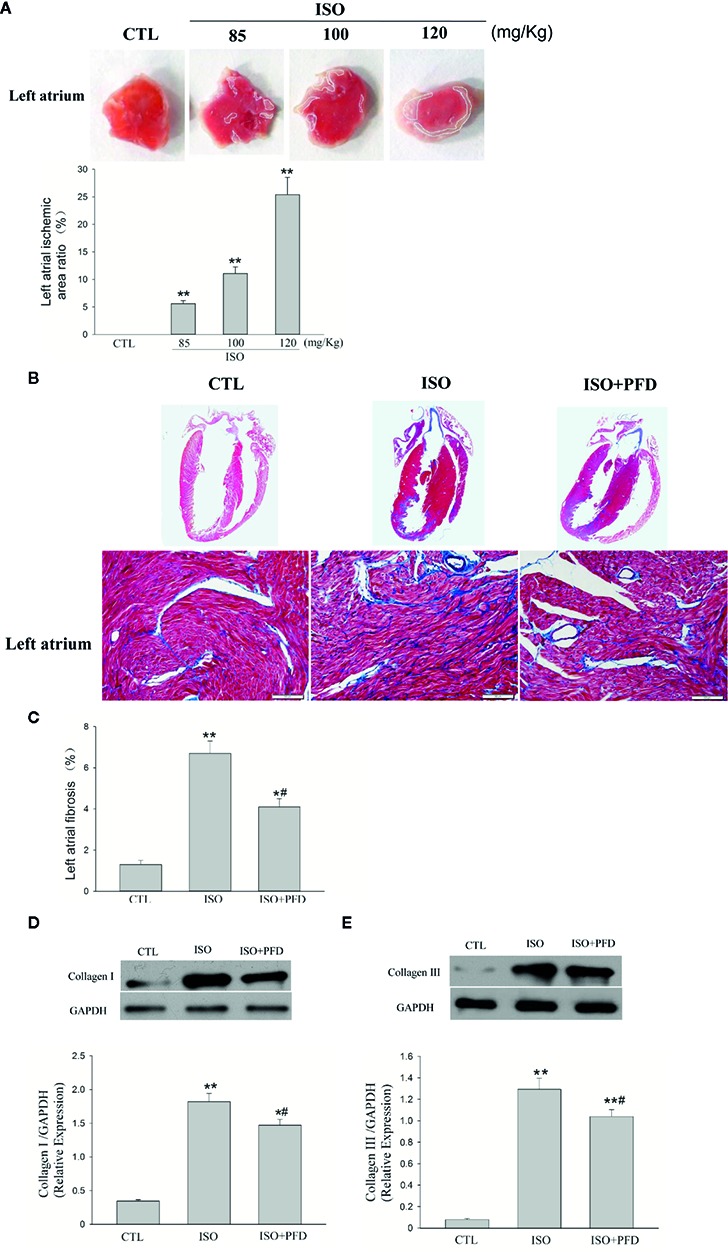
Isoproterenol (ISO) causes left atrial ischemia and fibrosis in rats. **(A)** Representative images of left atrium by 2, 3, 5-triphenylteyltetrazolium chloride (TTC) staining. Red-colored regions in the TTC stained sections indicate nonischemic areas; pale-colored regions indicate ischemic portions of the heart. Quantification of ischemic area/total area in the left atrium (*n* = 5 rats/group). **(B)** Representative images for myocardial fibrosis of the whole heart (Masson’s trichrome staining, which stains fibrosis blue and viable muscle red; scale bar: 100 µm). **(C)** Quantitation of left atrial fibrosis; ISO increased fibrosis-positive areas in the left atrium. **(D)** Western blot analysis of collagen I protein expression. ISO increased the protein levels of collagen I in the left atrium (*n*=5 rats/group). **(E)** Western blot analysis of collagen I protein amounts. ISO increased the protein levels of collagen III in the left atrium (*n*=5 rats/group). *P < 0.05, **P < 0.01 versus control (CTL) group; ^#^ *P* < 0.05 versus ISO group.

Representative illustrations of myocardial injury after TTC staining are shown in **Figure 1A**. CTL rats exhibited major portions stained positively, indicating tissue viability. There was little or zero percent of infract, however, the ISO group showed some unstained areas in the atrium. It is concentration-dependent increased in ISO group. The infarct size was significantly larger in 120 mg/kg group (25.4% ± 3.1%) than two lower dose groups. Masson’s trichrome staining of heart sections confirmed that ISO injection with 120 mg/kg (ISO group) resulted in increased fibrosis in the left atrium 5 weeks later (**Figure 1B**). The fibrotic area was overtly decreased in the ISO+PFD group compared with the ISO group (**Figure 1C**). Type I and III collagen was detected by western blot to further assess fibrosis (**Figures 1D, E**). ISO administration resulted in increased deposition of type I (*P*< 0.05, **Figure 1D**) and III (*P* < 0.05, **Figure 1E**) collagen in the left atrium. Antifibrosis drug PFD treatment significantly reduced such deposition (*P* < 0.05).

### ISO Increases Susceptibility to AF Five Weeks After Injection

**Figure 2:**
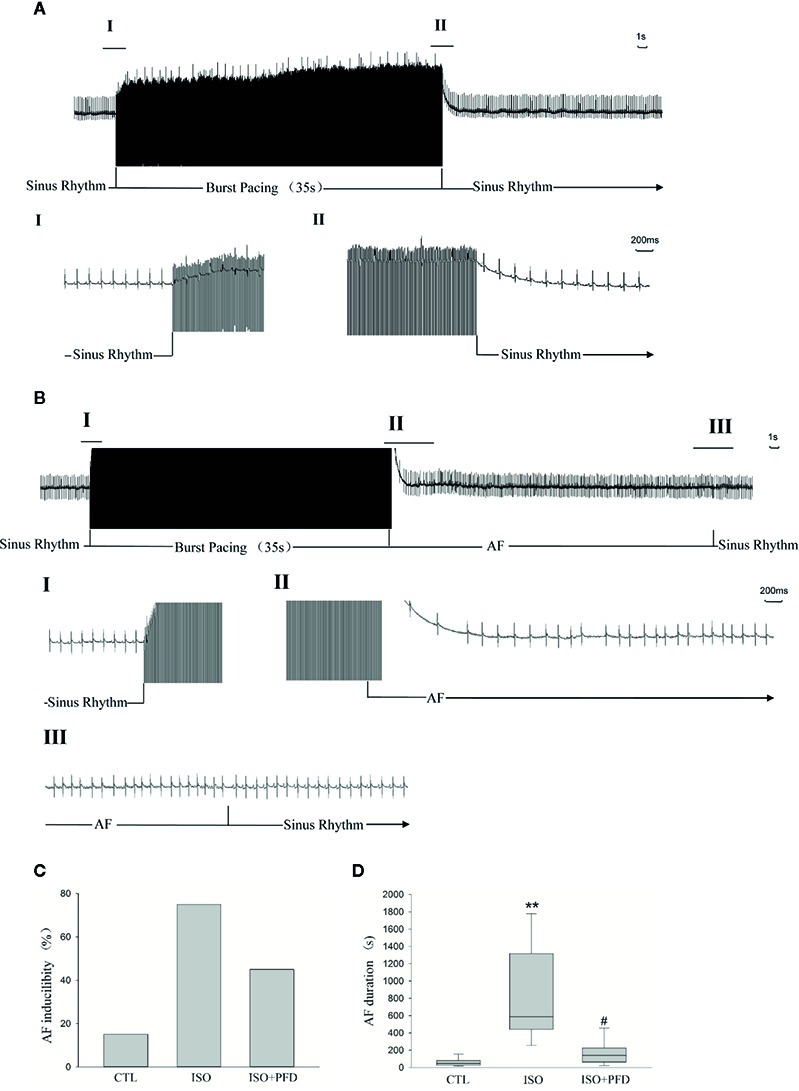
Isoproterenol (ISO) increases atrial fibrillation (AF) inducibility and duration in rats 5 weeks after injection. **(A)** Representative noninduced AF episode. After termination of the burst, the rat also displayed sinus rhythm (II). **(B)** Representative induced AF episode. Before the burst (I), the rat was in the sinus rhythm. After termination of the burst (II), the rat displayed an irregular atrial rhythm with an irregular ventricular response. After seconds (III), the AF episode stopped spontaneously and the sinus rhythm resumed. **(C)** ISO increases AF inducibility in rats (*n*=20 rats/group). **(D)** ISO increases AF duration (*n*=20 rats/group). **P < 0.01 versus control (CTL) group; ^#^ *P* < 0.05 versus ISO group.

In the 7 days after ISO subcutaneous injection, total mortality (10/50, 20%) was higher than in CTL rats (no death). Spontaneous episodes of AF were not observed throughout the induced episodes. **Figures 2A**, B show representative examples of non-AF and AF ECG. **Figure 2B** shows a representative example of induced AF electrocardiogram. AF occurred after induction termination by transesophageal programmed electrical stimulation (**Figure 2B II**). After seconds, the AF episode spontaneously stopped, and the sinus rhythm resumed. Susceptibility to AF in ISO treated rats (15/20, 75%) was significantly higher than that of CTL rats (3/20, 15%, **Figure 2C**). Treatment with PFD resulted in significantly decreased inducibility to 45% (9/20, *P* < 0.01). The mean AF episode duration was obviously longer in ISO treated rats compared with CTL animals (*P* < 0.01, **Figure 2D**). PFD treatment significantly decreased AF duration (*P* < 0.05, **Figure 2D**).

### Electrocardiographic Findings

**Figure 3:**
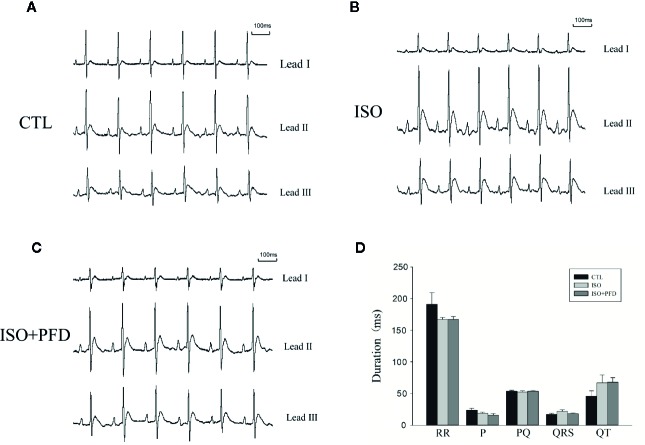
Surface ECGs in rats 5 weeks after injection. **(A)** Representative ECG in the control (CTL) group. Einthoven Leads: Leads I, II, and III. **(B)** Representative ECG in the isoproterenol (ISO) group. **(C)** Representative ECG in the ISO+ pirfenidone (PFD) group. **(D)** Bar graph indicates ECG intervals and durations. There were no significant differences in ECG parameters in rats 5 weeks after ISO injection (*n*=20 rats/group).

Three-lead electrocardiograms were recorded in anesthetized rats 5 weeks after injection. **Figure 3** depicts representative examples of ECGs. Surface ECG parameters were summarized in **Figure 3D**. P duration, RR interval, PQ interval, QRS, and QT durations were not significantly different among the three groups (*P* > 0.05).

### ISO Decreases Cardiac Function

**Figure 4:**
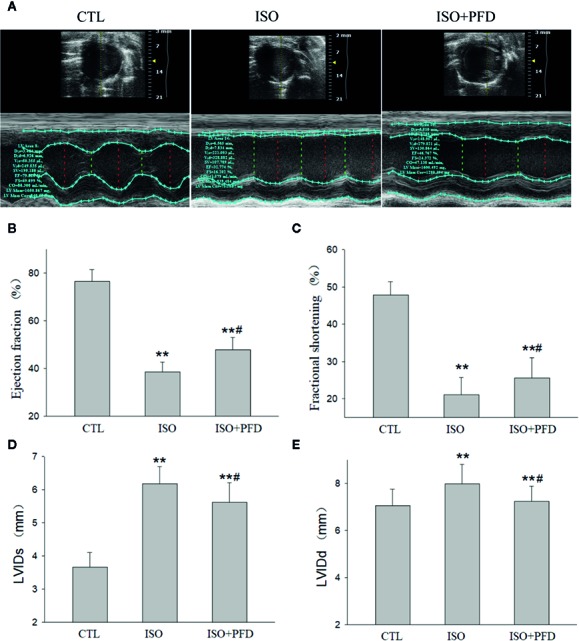
Isoproterenol (ISO) decreases cardiac function. **(A)** Representative M-mode echocardiogram in rats 5 weeks after ISO injection. **(B)** Bar graph indicates EF (*n*=20 rats/group). **(C)** Bar graph indicates FS (*n* = 20 rats/group). **(D)** Bar graph indicates left ventricular internal dimensions during systole (LVIDs) (*n* = 20 rats/group). **(E)** Bar graph indicates left ventricular internal dimensions during diastole LVIDd (*n*=20 rats/group). **P < 0.01 versus control (CTL) group, ^#^ *P* < 0.05 versus ISO group.

Five weeks after injection, echocardiography showed clear anterior wall motion abnormality (**Figure 4A**). As shown in **Figures 4B, C**, the ISO group had reduced EF (76.5% ± 4.9% vs. 38% ± 4.1%, *P* < 0.05) and FS (47.8% ± 3.6% vs. 21.1% ± 4.7%, *P* < 0.05) compared with the CTL group. Both FS and EF in the PFD group were increased compared with values obtained for the ISO group (*P* < 0.05). Both LVIDs (*P* < 0.01, **Figure 4D**) and LVIDd (*P* < 0.01, **Figure 4E**) were elevated in the ISO group, but decreased after PFD treatment (both *P* < 0.05).

### ISO Increases LA Conduction Heterogeneity

**Figure 5:**
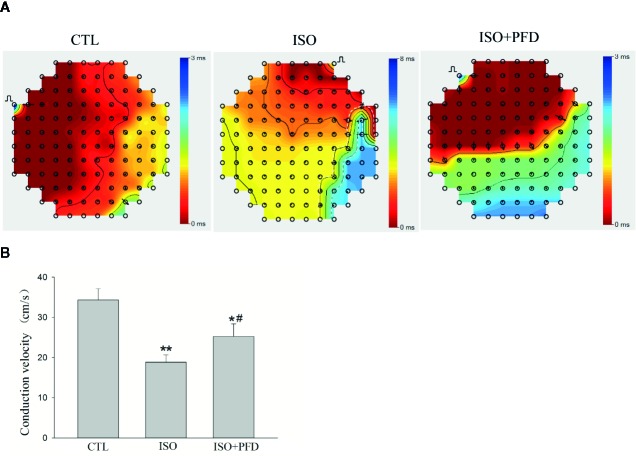
Isoproterenol (ISO) increases left atrial conduction heterogeneity in rats. **(A)** Representative isochronous maps in the left atrium as obtained by multielectrode array (MEA) recording. Areas of isochronal crowding were found in the ISO group. The degree of crowding decreased in pirfenidone (PFD)-treated rats. Conduction was more heterogeneous in the ISO group compared with the control (CTL) group. **(B)** Bar graph indicates CV (*n*=5 rats/group). *P < 0.05, **P < 0.01versus CTL group; ^#^ *P* < 0.05 versus ISO group.

LA surface conduction was measured using a 120-electrode MEA. Isochronal maps clearly showed a large zone of conduction blockage, which could block wave propagation in the ISO group (**Figure 5A**). The activation located distally propagated to the block zone. There was no or limited conduction block zone in the CTL and PFD-treatment groups. Compared with the CTL and PFD-treatment groups, the ISO group showed more heterogeneous conduction. CV in the ISO group was significantly lower than that of the CTL group (**Figure 5B**, *P* < 0.01). Compared with the ISO group, PFD administration increased the LA CV (*P* < 0.05). PFD improved the LA CV and homogeneity.

### ISO Induces Myofibroblast Differentiation

**Figure 6:**
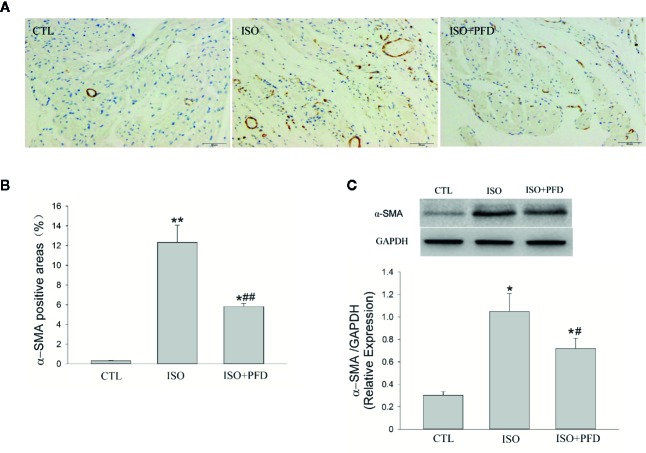
Isoproterenol (ISO) induces myofibroblast differentiation in rats. **A**. Representative images showing the expression and distribution of the myofibroblast-specific marker α-SMA (Immunochemistry, scale bar: 50 µm) in the left atrium. **(B)** Quantitation of α-SMA positive areas. ISO increased α-SMA positive area (*n*=5 rats/group). **(C)** Western blot analysis of α-SMA protein expression. ISO increased the protein levels of α-SMA (*n*=5 rats/group). *P < 0.05, **P < 0.01 versus control (CTL) group, ^#^ *P* < 0.05, ^##^ *P* < 0.01 versus ISO group.

To assess the effects of ISO on fibroblast differentiation into myofibroblasts, immunohistochemistry was performed to detect α-SMA levels (**Figure 6A**). The results showed that ISO induced α-SMA expression in the left atrium (*P* < 0.05, **Figure 6B**). This effect was further validated by α-SMA protein expression levels. Compared with CTL rats, ISO treatment resulted in increased α-SMA protein levels (*P* < 0.05, **Figure 6C**). PFD reduced α-SMA-positive areas and protein amounts.

### ISO Induces Atrial Ischemic Infarction by Increasing the Heart Rate and Reduces Blood Pressure

**Figure 7:**
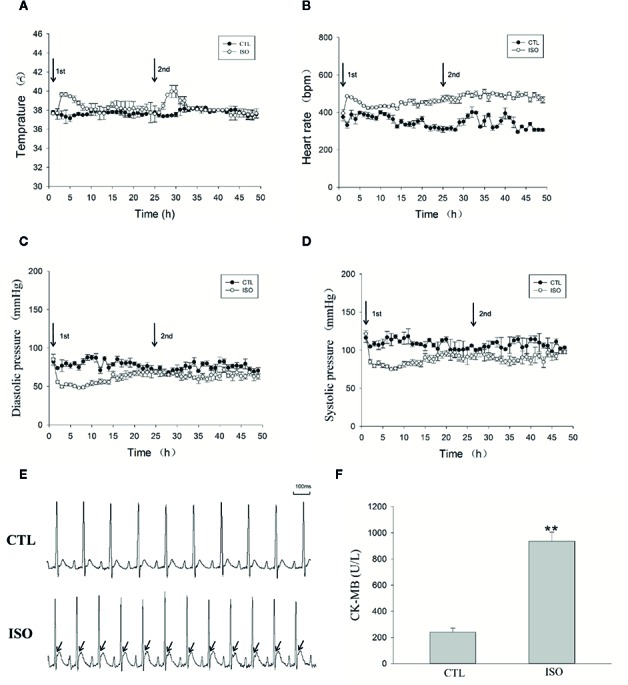
Isoproterenol (ISO) induces left atrial ischemia by increasing the heart rate and reducing blood pressure. An implantable telemetry transmitter was used for the continuous monitoring of temperature, ECG, and blood pressure. **(A)** Temperature analysis during the 48-h following ISO injection. **(B)** Heart rate during the 48-h following ISO injection. **(C)** Diastolic pressure during the 48-h following ISO injection. **(D)** Systolic pressure during the 48-h following ISO injection. **(E)** Representative ECG after injection of ISO. **(F)** Bar graph indicates creatinine kinase-MB (CK-MB) changes after ISO injection. **P < 0.01 versus CTL group.

**Figure 7A** telemetry transmitter recording showed that ISO injection resulted in at least 6–7 h increase in temperature. The heart rate was also increased substantially after treatment with ISO (**Figure 7B**). However, injection of 0.9% saline had not obviously changes in CTL rats. Heart rates before ISO injection were similar in both groups. The maximum heart rate change was from 395.2 ± 21.5 to 486.6 ± 2.9 bpm 1 h after first ISO injection, which increased oxygen consumption. **Figures 7C, D** show mean blood pressures 48 h after injection. Abdominal aortic arterial pressure decreased from 121.4 ± 4.3 to 84.6 ± 2.8 mmHg and 85 ± 6.7 to 55.7 ± 2 mmHg for systolic and diastolic blood pressures 1 h after first ISO injection. The blood pressure reduction continued for about 20 h. Before the second injection, arterial pressure in ISO treated rats was close to that of CTL rats. The second ISO injection caused further decrease in arterial pressure in rats. Two injections of 0.9% saline in CTL rats had no obvious effects on arterial pressure. ISO treatment resulted in significantly elevated ST-segment (**Figure 7E**) and increased CK-MB levels (*P* < 0.01, **Figure 7F**). These data indicate that ISO caused myocardial ischemic infarction by increasing heart rate, and decreasing diastolic and systolic blood pressures.

## Discussion

Cardiac fibrosis in the left atrium is an important arrhythmogenic substrate for AF. This study showed that: (1) ISO increased AF inducibility and extended its duration in rats 5 weeks after injection; (2) ISO increased LA fibrosis and LA conduction heterogeneity in rats 5 weeks after injection; (3) ISO injection induced atrial ischemic infarction by increasing the heart rate and decreasing coronary flow due to a significant drop in blood pressure; (4) Antifibrosis drug PFD decreased AF occurrence in rats 5 weeks after ISO injection by reducing LA fibrosis. Taken together, these results suggested that ISO could increase LA fibrosis and AF susceptibility 5 weeks after injection by inducing atrial ischemic injury. Rats with ISO-induced LA fibrosis may be used as a model in AF research.

Myocardial ischemia refers to the pathological state of reduced oxygen supply and residual metabolites caused by decreased blood perfusion, and reflects an imbalance between myocardial oxygen supply and demand (Heusch, 2016). In many circumstances, myocardial ischemia results from the combined effects of increased oxygen demand and reduced amounts of oxygen. ISO, a systemic β-adrenergic receptor agonist, is associated with marked ventricular myocardial ischemia (Patel et al., 2016; Song et al., 2016), hypertrophy (Zhang et al., 2015) and fibrosis (Ma et al., 2017). Merino reported that ISO increases both atrial frequency and contractility (Merino et al., 2015), suggesting that ISO may affect the atrium. In the present study, high doses of ISO injected at an interval of 24 h induced a variety of myocardial ischemic injury phenomena, such as ST-segment elevation and increased plasma CK-MB, by increasing the heart rate and decreasing diastolic and systolic blood pressures (also accompanied by a decrease in coronary flow) for hours. TTC staining showed large areas of ischemia zone in the atrium after injection. Masson’s trichrome staining also showed large fibrotic areas in the left atrium 5 weeks after injection. These results strongly suggested that ISO injection induces atrial ischemic infarction by promoting imbalance between increased myocardial oxygen demand and reduced coronary blood supply. In clinic, atrial infarction is rarely diagnosed before death because of its characteristic subtle and nonspecific electrocardiographic findings (Lu et al., 2016). Atrial infarction has been observed in 17% of MI patients in a large postmortem study. In addition, increased risk of atrial tachyarrhythmia has been observed in patients with atrial infarction. Studies on atrial ischemic infarction in animal models (dogs, sheep, and pigs) by left circumflex coronary artery ligation also suggested that experimental atrial ischemia could create a substrate for AF maintenance (Aguero et al., 2017a).

High doses of ISO stimulate myocardial ischemia, hypoxia, necrosis, and fibroblastic hyperplasia, which are strongly similar to local myocardial damage and acute myocardial infarction (Qu et al., 2020). After myocardial injury, various peptide growth factors stimulate fibroblasts to migrate into the wound site and proliferate to reconstitute various connective tissue components (Dobaczewski et al., 2012; Lajiness and Conway, 2014). Otherwise, ISO could directly induce cardiac fibroblast proliferation and collagen synthesis *in vivo* (Sun et al., 2015). A critical event in the process is fibroblast differentiation into active-phenotype myofibroblasts (Honda et al., 2013; Mack and Yanagita, 2015). This results in functional changes, including increased proliferation, altered release of signaling molecules, and extracellular matrix deposition (Vasquez et al., 2011). In wound healing these cells provide additional extracellular collagen fiber deposition, which strengthens the injured tissue. However, when myofibroblasts persist in injured areas and continue to function, this helpful response becomes harmful, leading to progressive fibrosis (Davis and Molkentin, 2014). The present data showed that heart myofibroblasts persisted in atrial infarct scars, which induced large atrial fibrosis areas 5 weeks after ISO injection. The present findings corroborated Aguero *et al.*, who assessed atrial fibrosis changes in dogs with atrial infarction by left circumflex coronary artery ligation (Aguero et al., 2017b).

Cardiac fibrosis refers to a variety of quantitative and qualitative changes in the interstitial myocardial collagen network, and occurs in response to cardiac ischemic insults, systemic diseases, drugs, or other harmful stimuli; it alters myocardial architecture, promoting the development of cardiac dysfunction and arrhythmias, and influencing the clinical course and outcome of related-diseases. With the development of noninvasive methods, such as the late gadolinium–enhanced cardiac magnetic resonance (LGE-CMR) imaging technology, LA fibrosis is considered the hallmark of structural remodeling in AF and the substrate for AF maintenance (Azadani et al., 2017). Experimental studies have provided convincing evidence that fibrotic transformation of the atrium results in altered atrial conduction, increasing impulse propagation anisotropy. Heterogeneous atrial tissue is more susceptible to reentry, leading to conduction blockage in regions with high conduction anisotropy. In LA appendages from patients undergoing antiarrhythmic surgery for AF, the optical mapping technology found conduction abnormalities with different longitudinal conduction velocities in different regions (Arroja et al., 2016). In the present study, conduction abnormalities were also found in the left atrium, with elevated AF inducibility 5 weeks after ISO injection in rats. The mean CV in the ISO group was significantly lower than that of CTL rats. These results suggested that ISO increases susceptibility to AF by enhancing LA fibrosis and conduction heterogeneity 5 weeks after injection.

In recent years, several animal models with increased atrial interstitial fibrosis have been described with high vulnerability to AF. Spontaneously hypertensive rats develop a substrate for AF *via* increased LA interstitial fibrosis (Lau et al., 2013). Left coronary artery ligation in rats leads to heart failure, with atrial dilatation, atrial fibrosis and AF promotion (Cardin et al., 2012; Ma et al., 2018a; Ma et al., 2018b). A transgenic mouse with TGF-β1 overexpression and selective atrial fibrosis has increased AF inducibility. These animal models are widely used in studies exploring mechanisms and pharmacological therapeutics for AF (Zhang et al., 2013; Zhang et al., 2014; Ma et al., 2018a). In the present study, ISO increased susceptibility to AF by enhancing LA fibrosis and conduction heterogeneity 5 weeks after injection. PFD is one of two approved therapies for the treatment of idiopathic pulmonary fibrosis. Basic and clinical evidence suggests PFD may slow or inhibit the progressive fibrosis after tissue injuries. In vitro studies have shown that PFD can attenuate the proliferation and activation of fibroblasts and the expression of profibrotic factors (Shi et al., 2011). PFD significantly reduced arrhythmogenic atrial fibrosis and AF vulnerability in congestive heart failure canines (Lee et al., 2006). In this study, we used PFD (300 mg/kg, the usual dose) as a positive control drug to test the ISO model. Pirfenidone was given 1 week after ISO injection for 4 weeks to avoid impairing early repairs, according to the Nguyen study (Nguyen et al., 2010). After four weeks of administration, PFD decreased AF inducibility (45% in the ISO+PFD group vs. 75% in the ISO group) and LA fibrosis area caused by ISO (*P* < 0.05), close to the effects of PFD or other antifibrotic drugs in the myocardial infarction model induced by ligation of the left anterior descending coronary artery (Ma et al., 2018c; Qiu et al., 2018). PFD also improved the cardiac function, CV and reduced myofibroblasts differentiation. The effects of PFD in this ISO model were close to other antifibrosis drugs in other animal models (Wang et al., 2013; Beiert et al., 2017; Ma et al., 2018c). These results indicate that ISO-induced LA fibrosis rats may be used as a model in AF research. Compared with other animal models, the ISO model has the advantages of low cost, easy operation and good repeatability (Allawadhi et al., 2018). The effects of ISO on other vulnerable substrates for AF apart from fibrosis are unclear and need further investigation.

## Conclusions

The present study showed that high-dose ISO induces atrial ischemic infarction in rats. Five weeks after injection, ISO increased LA fibrosis and LA conduction heterogeneity, ultimately leading to increased susceptibility to AF in rats. Rats with ISO-induced LA fibrosis may be used as a model in AF research.

## Data Availability Statement

All datasets generated for this study are included in the article/supplementary material.

## Ethics Statement

The animal study was reviewed and approved by Animal Care Commitee of Guangdong Provincial Hospital of Chinese Medicine.

## Author Contributions

SM and JM conceived the study, designed, performed, and analyzed the experiments, carried out the data collection and wrote the paper. QT and CZ carried out the data collection. QC and WL coordinated the study and revised the paper. All authors reviewed the results and approved the final version of the manuscript.

## Funding

This work was supported by grant from Guangdong Basic and Applied Basic Research Foundation (No.2019A1515010808), Natural Science Foundation of Guangdong Province (No.2017A030313888, No.2016A030313634), TCM Science and Technology Foundation of Guangdong Provincial Hospital of Chinese Medicine (No.YN2018MJ02), and Guangzhou science and Technology Foundation (No.201607010364).

## Conflict of Interest

The authors declare that the research was conducted in the absence of any commercial or financial relationships that could be construed as a potential conflict of interest.
